# Supplementary figures and images for: Increased preference for lysine over arginine in spike proteins of SARS-CoV-2 BA.2.86 variant and its daughter lineages
Source: PLoS One. 2025 Apr 7;20(4):e0320891. doi: 10.1371/journal.pone.0320891 (PMC11975073; doi:10.1371/journal.pone.0320891)

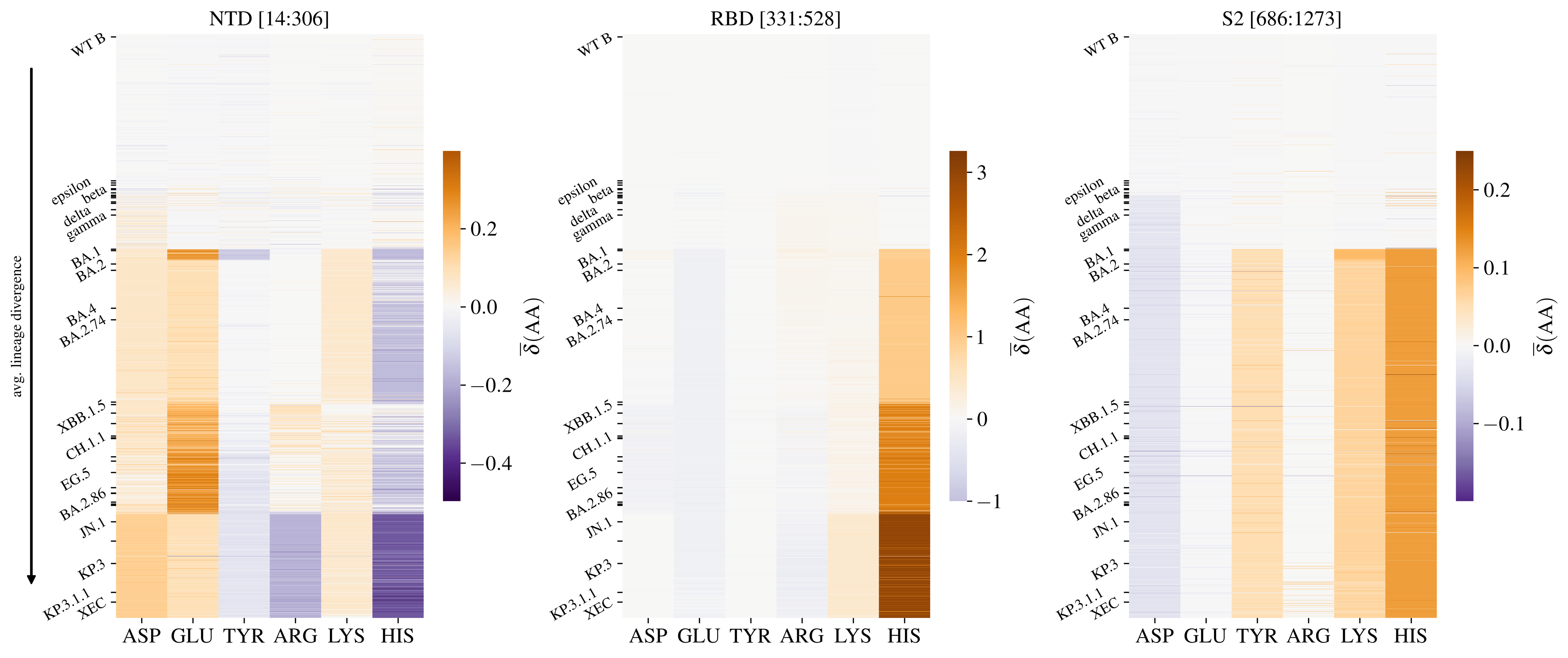

Supplement: S1 Fig — Shown are the changes in the N-terminal domain (NTD), receptor-binding domain (RBD), and S2 domain of the spike protein. Region boundaries are taken from Ref. [92] and are determined with respect to the WT SARS-CoV-2 spike protein sequence. Panels show the average change δ¯(AA) [Eq (1)] in the number of negatively charged (ASP, GLU, and TYR) and positively charged (ARG, LYS, and HIS) amino acids of 2665 different SARS-CoV-2 lineages compared to WT lineage B. Lineages are ordered according to their (average) divergence from WT B. (TIF) [file pone.0320891.s001.tif]

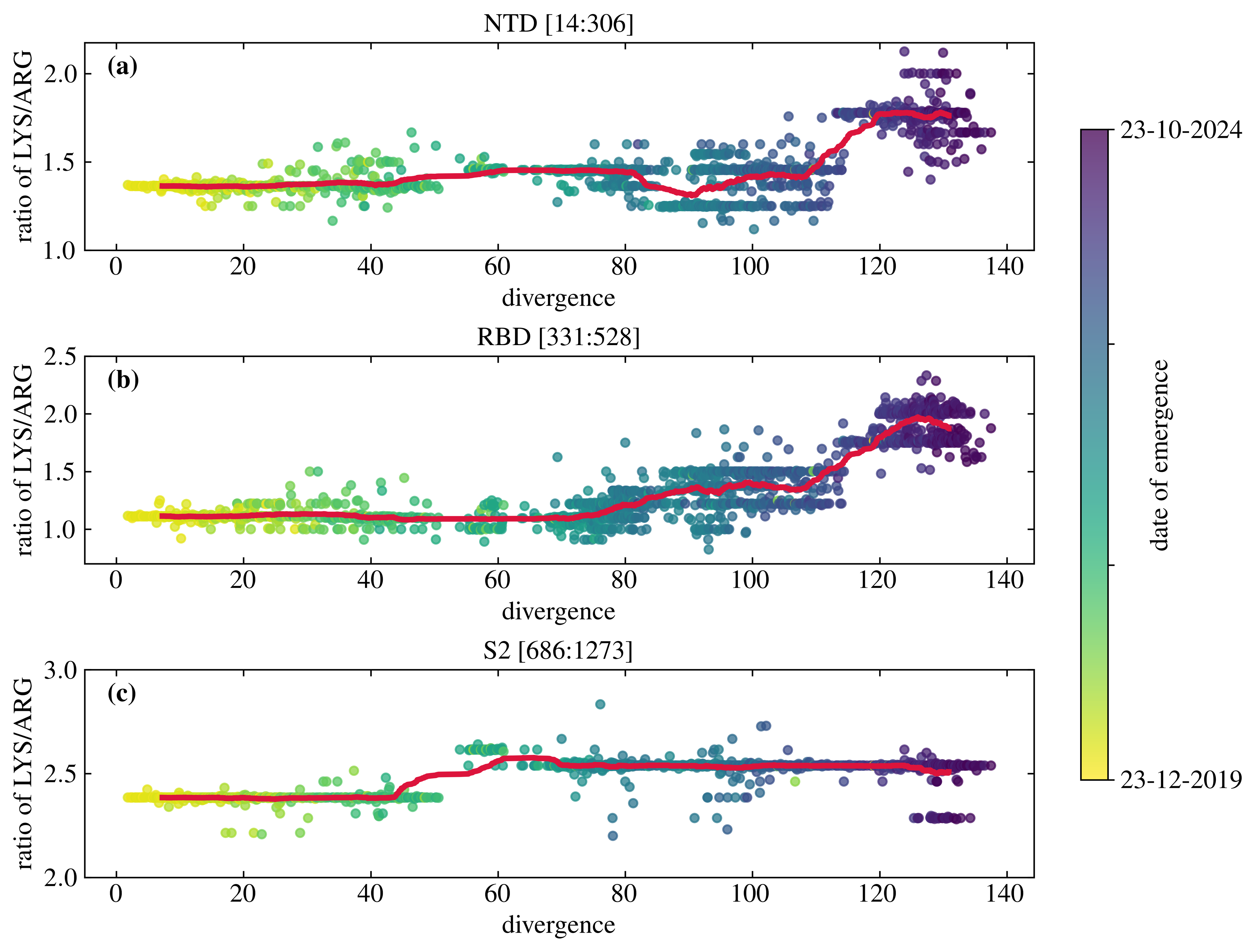

Supplement: S2 Fig — Shown are the changes in the LYS/ARG ratio in the (a) NTD, (b) RBD, and (c) S2 domain as a function of the (average) lineage divergence. Each point in the panels represents one of the 2665 different SARS-CoV-2 lineages analyzed. Thick lines show a rolling average of the LYS/ARG ratio with lineage divergence (window size contains 100 lineages). (TIF) [file pone.0320891.s002.tif]

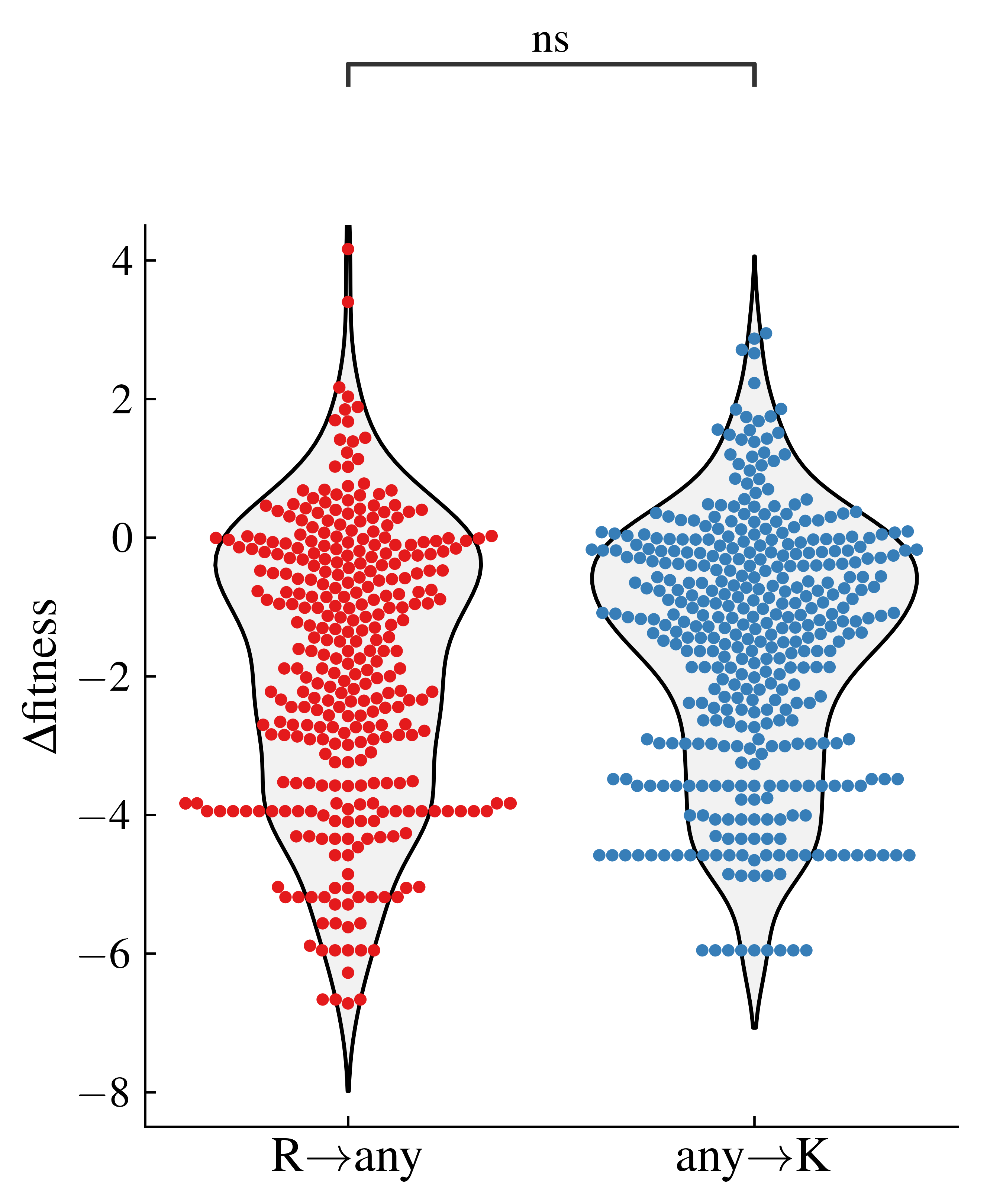

Supplement: S3 Fig — Viral fitness is estimated as the logarithm of actual vs. observed mutation counts in publicly available SARS-CoV-2 sequences as of March 2023; data are taken from Ref. [10]. Compared are the mutations in the spike protein from arginine to any amino acid (R → any) and from any amino acid to lysine (any → K). These two types of mutations do not differ significantly in their effect on viral fitness (Brunner-Munzel test, p = 0 . 06). (TIF) [file pone.0320891.s003.tif]

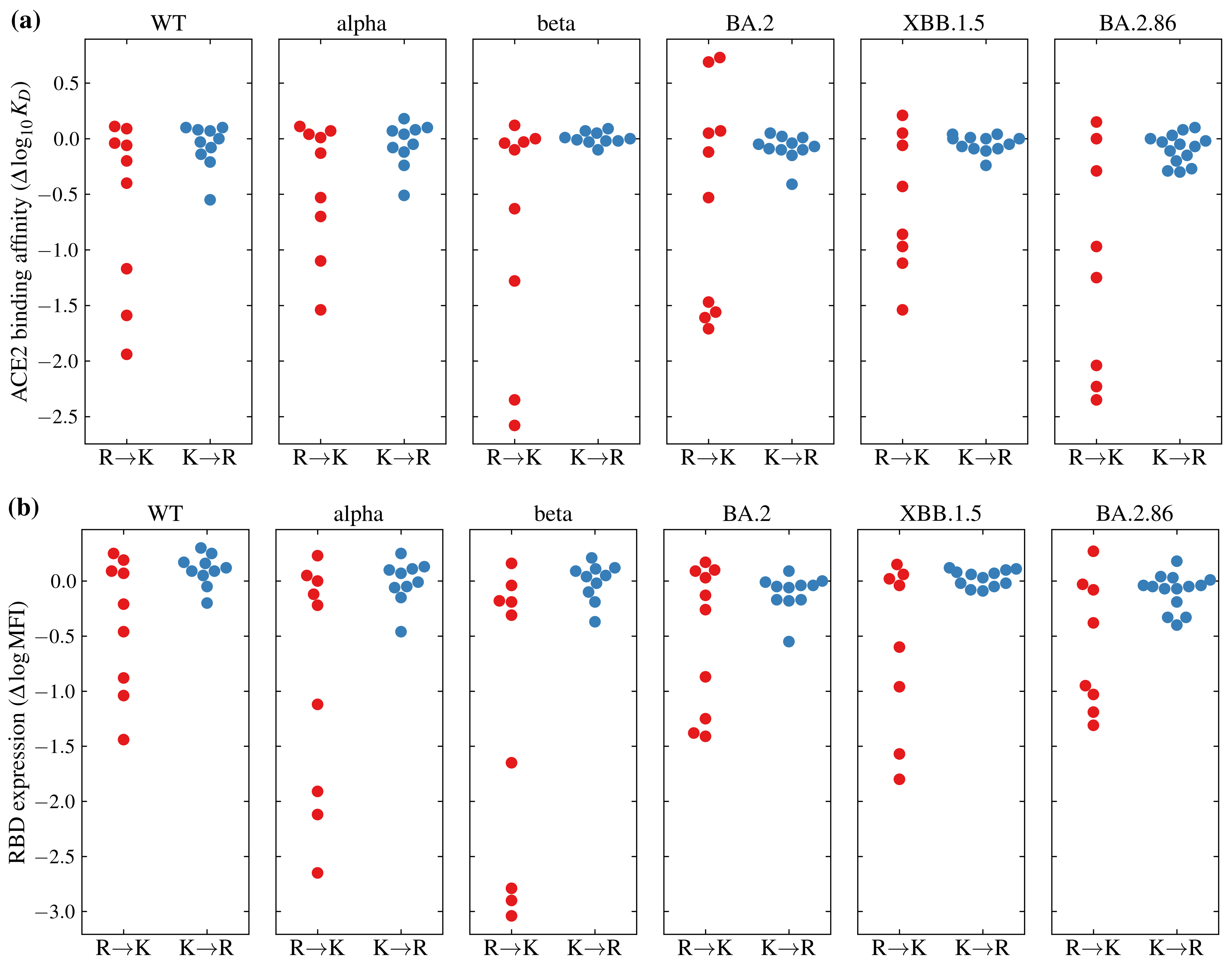

Supplement: S4 Fig — Shown are the effects of spike protein mutations from ARG to LYS and vice versa on (a) ACE2 binding affinity and (b) RBD expression of select SARS-CoV-2 variants. Data are taken from Ref. [49]. (TIF) [file pone.0320891.s004.tif]

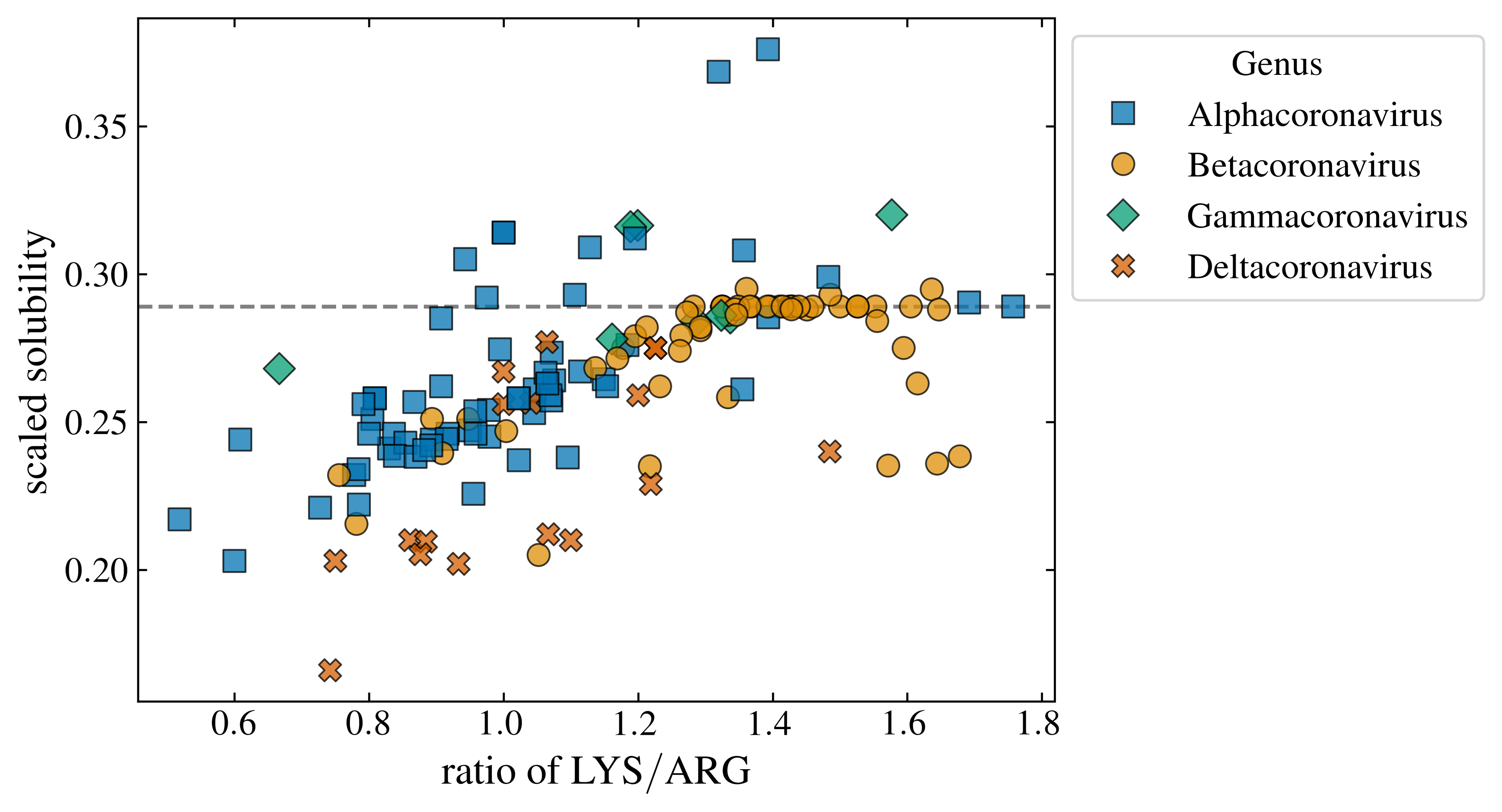

Supplement: S5 Fig — Dataset of viruses from the four genera of Orthocoronavirinae subfamily is the same as in Fig 4 in the main text. Scaled solubility was predicted from spike protein sequences using protein-sol [74]. Dashed line shows the predicted scaled solubility of 0 . 289, which is predicted to be the same for the entire dataset of SARS-CoV-2 spike protein sequences. (TIF) [file pone.0320891.s005.tif]
